# Structure-based cross-docking analysis of antibody–antigen interactions

**DOI:** 10.1038/s41598-017-08414-y

**Published:** 2017-08-15

**Authors:** Krishna Praneeth Kilambi, Jeffrey J. Gray

**Affiliations:** 1 Department of Chemical and Biomolecular Engineering, The Johns Hopkins University, Baltimore, MD 21218 USA; 2 Biogen, Cambridge, MA 02142 USA

## Abstract

Antibody–antigen interactions are critical to our immune response, and understanding the structure-based biophysical determinants for their binding specificity and affinity is of fundamental importance. We present a computational structure-based cross-docking study to test the identification of native antibody–antigen interaction pairs among cognate and non-cognate complexes. We picked a dataset of 17 antibody–antigen complexes of which 11 have both bound and unbound structures available, and we generated a representative ensemble of cognate and non-cognate complexes. Using the Rosetta interface score as a classifier, the cognate pair was the top-ranked model in 80% (14/17) of the antigen targets using bound monomer structures in docking, 35% (6/17) when using unbound, and 12% (2/17) when using the homology-modeled backbones to generate the complexes. Increasing rigid-body diversity of the models using RosettaDock’s local dock routine lowers the discrimination accuracy with the cognate antibody–antigen pair ranking in bound and unbound models but recovers additional top-ranked cognate complexes when using homology models. The study is the first structure-based cross-docking attempt aimed at distinguishing antibody–antigen binders from non-binders and demonstrates the challenges to address for the methods to be widely applicable to supplement high-throughput experimental antibody sequencing workflows.

## Introduction

Antibody–antigen interactions are an important component of our immune response to pathogens^1^, and understanding the structural basis of antibody–antigen interactions can help in designing more potent therapeutics. Recent experimental advances^2^ have enabled high-throughput sequencing studies of antibody repertoires in multiple organisms^3–5^, including perturbation effects of various antigens on the repertoire sequence frequencies^6–8^. Since antibody repertoire sizes for an individual range from 10^3^ for zebrafish to 10^10^ for humans^9, 10^, computational approaches represent a practical structure-based method to study antibody specificity and selectivity. To understand the driving forces behind the generation and maturation of these antibody sequences, we need not just structural models, but knowledge of the antigen/epitope pairings and models of the bound antibody–antigen complexes. In this paper, we present a computational cross-docking study to discriminate binders from non-binders by identifying native antibody–antigen interaction pairs among cognate and non-cognate complexes. We also discuss the major remaining challenges for the methods to be useful to support high-throughput next-generation sequencing (NGS) pipelines or to support individual studies on particular antibody–antigen complexes of interest.

Cross-docking study efficacy relies on the accuracy of binding energy estimates. A team of researchers collected experimental binding affinity measurements for 179 protein–protein complexes as a benchmark dataset for training computational algorithms^11, 12^. Kastritis and Bonvin tested the affinity prediction ability of nine of the standard score functions used in the leading docking algorithms and found all score functions correlated poorly with the experimental results^13^. Some score functions were able to broadly classify weak, medium and strong binders, but the standard deviations of the predicted binding energies were wider than the energy gap between the three categories. The ability to distinguish binding from non-binding interfaces was also tested in Critical Assessment of PRediction of Interactions (CAPRI) through a challenge to predict successful high-affinity binders from a set of designed protein–protein interfaces and distinguishing natural interfaces from unsuccessful Rosetta-designed interfaces^14^. Both these challenges were difficult, and no computational method was able to identify the design responsible for the successful binder.

Antibodies constitute the most important class of therapeutic biologics. Accurate binding estimates can aid development of *in silico* screening methods to pick a potential list of epitope-diverse antibodies during animal immunization NGS campaigns, and help design better-behaving therapeutic antibodies with minimal off-target activity. Unfortunately, estimation of absolute binding free energies for antibody–antigen complexes is challenging due to the inaccuracies in the computational free energy calculations. Additionally, since all antibodies share the immunoglobulin fold with sequence variability primarily in the complementarity determining regions (CDRs), domain or sequence-similarity based binding predictors are not reliable. So accurate biophysics-based cross-docking methods aimed at identifying potential antibody–antigen interaction partners are of immense value. Previous cross-docking attempts^15, 16^ on a dataset of diverse protein–protein complexes found the prediction of antibody–antigen interaction pairs to be especially difficult.

In this paper, we present a cross-docking study to discriminate antibody–antigen binders from non-binders using a dataset of 17 complexes with both bound and unbound antigen structures available. We first generate a dataset of cognate and non-cognate complexes and test the RosettaDock score function for identification of the correct antibody–antigen interaction partners. We evaluate the effects of antibody backbone accuracy on the partner predictions using antibody bound, unbound structures, and RosettaAntibody^17^ generated homology models. We also demonstrate the effects of increasing diversity of the generated cognate and non-cognate models on prediction accuracy. To our knowledge, our work is the first structure-based cross-docking study focused on distinguishing antibody–antigen binders from non-binders.

## Results

Antibody-antigen complexes are well suited for cross-docking based affinity calculations because antibodies can be superposed using their framework regions onto different antibodies binding their cognate epitopes. We assembled a test set of 17 antibody–protein-antigen pairs from the affinity benchmark set^11^ for which both bound and unbound antigen structures are known (Table 1). Eleven of these complexes also have both bound and unbound structures available for the antibody, and the remaining six have solved bound antibody structures only.

**Table 1:** Antibody–antigen complexes used for cross-docking.

No.	Antibody	Antigen	Complex PDB	Antibody PDB	Antigen PDB	Expt. Kd (nM)
1	Fab B02C11	Factor VIII domain C2	1IQD	1IQD*	1D7P	<0.014
2	Fab N10	Staphylococcal nuclease	1NSN	1NSN*	1KDC	<0.10
3	Fab D3H44	Tissue factor	1JPS	1JPT	1TFH	0.10
4	FabF10.6.6	HEW lysozyme	1P2C	2Q76	3LZT	0.10
5	Fab BV16	Birch pollen antigen Bet V1	1FSK	1FSK*	1BV1	0.24
6	Fab E8	Cytochrome C	1WEJ	1QBL	1HRC	0.71
7	Fab HC19	Flu virus hemagglutinin	2VIR	1GIG	2HMG	1.00
8	Fab Hyhel63	HEW lysozyme	1DQJ	1DQQ	3LZT	2.80
9	Fab Jel42	HPr	2JEL	2JEL*	1POH	2.80
10	Fab 5g9	Tissue factor	1AHW	1FGN	1TFH	3.40
11	Fab A4.6.1	VEGF	1BJ1	1BJ1*	2VPF	3.40
12	Fv D1.3	HEW lysozyme	1VFB	1VFA	8LYZ	3.70
13	Fab NC41	Flu virus neuraminidase N9	1NCA	1NCA*	7NN9	8.30
14	Fv Hulys11	HEW lysozyme	1BVK	1BVL	3LZT	14
15	Fab 13B5	HIV-1 capsid protein p24	1E6J	1E6O	1A43	29
16	Fab 44.1	HEW lysozyme	1MLC	1MLB	3LZT	91
17	Fab HC19	Flu virus hemagglutinin T131I mutant	2VIS	1GIG	2VIU	4000

*In these six cases, the only antibody structure available is in the antigen-bound state.

We superposed each antibody onto the native antibody in 16 other antibody–antigen complexes, generating 289 total pairs to discriminate. The tightly packed interface residues in the native crystal complexes and the steric clashes in the non-cognate complexes generated using antibody superposition make the initial set trivial to separate. To construct a more realistic set, it is necessary to refine each candidate complex to (1) erase the memory of the crystal structure in cognate complexes, and (2) optimize the interface in non-cognate complexes. Furthermore, generation of an unbiased set of starting models is critical if the method is to be extendable to antibodies with no available experimental structures with the corresponding antigens. We used a customized version of the fixed-backbone RosettaDock high-resolution stage (“local refine”) to generate an ensemble of 50 cognate and non-cognate refined models for each antibody–antigen pair (see Methods for details).

**Figure 1:**
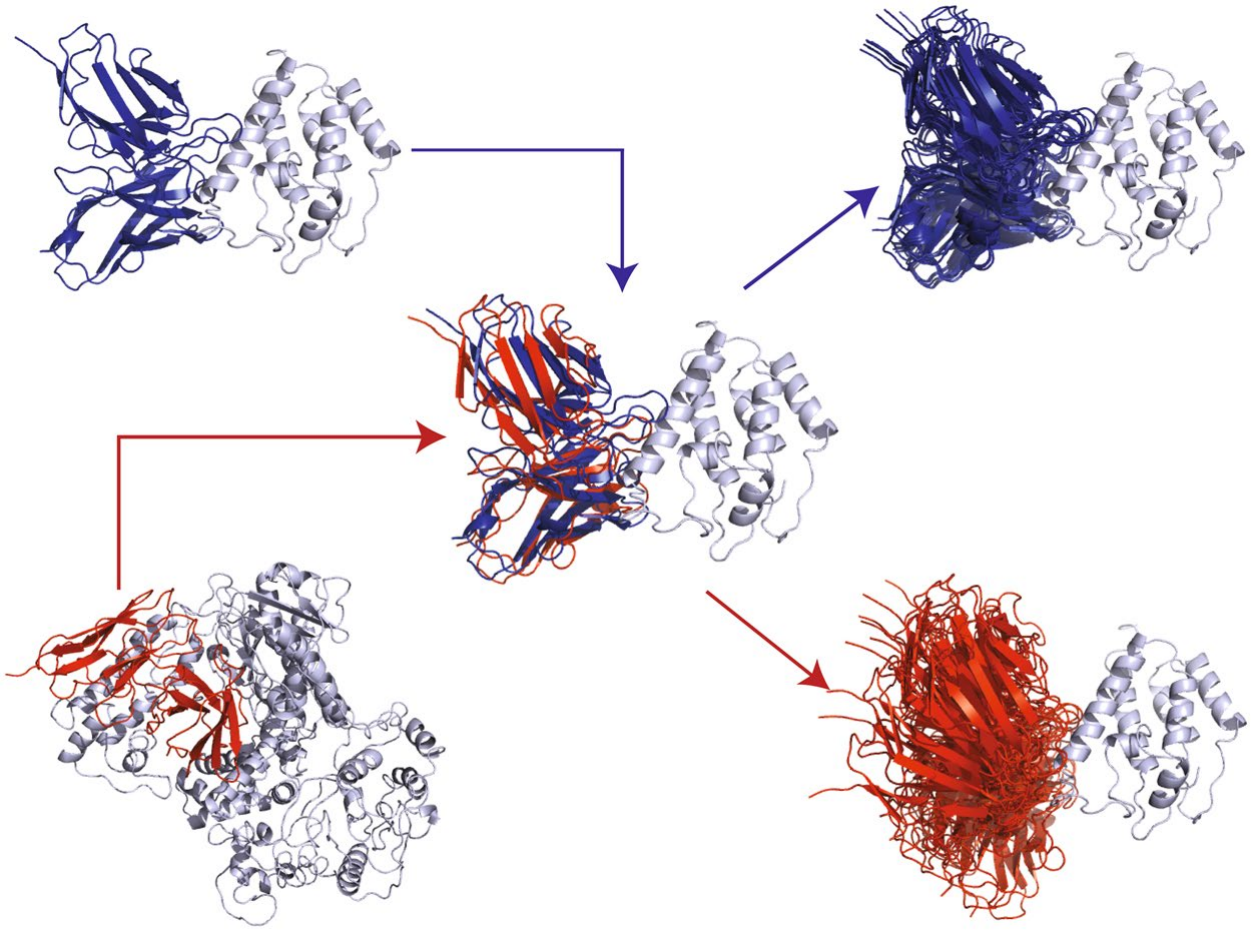
Generation of cognate and non-cognate antibody–antigen complexes. The starting structures for cognate models (blue) are the crystal complexes, while the starting non-cognate models are generated by superimposing the V_L_–V_H_ framework of the non-cognate antibody (red) on the cognate antibody in the crystal complex. A customized version of RosettaDock’s high-resolution stage is then used to generate an ensemble of models focused around the epitope for discrimination.

As shown in Fig. 1, the cognate models are refined starting from the crystal complex, while the non-cognate models are refined after superimposing the V_L_–V_H_ framework of the non-cognate antibody on the cognate antibody in the crystal complex. The starting antibody ligand-RMSDs between cognate and non-cognate antibodies average around 1.2–1.4 Å (Supplementary Fig. S1). The primary goal is to predict the correct antibody that pairs with each antigen. To test the effects of antibody backbone accuracy on the binding predictions, we generated cognate and non-cognate models using bound–bound (henceforth referred to as simply “bound”), unbound–unbound (“unbound”), and homology–unbound (“homology”) backbones, respectively, for the antibody–antigen complexes. About 35% (6/17) of the antibodies in the dataset lack unbound structures, so we used bound antibody backbones to generate their unbound cognate and non-cognate complex models. We used RosettaAntibody 3.0^18^ to generate the antibody models required for homology complexes (see Methods). Based on CDR RMSDs, most of the generated antibody models are approximately equidistant (1–3 Å) from the known bound and unbound conformations (Supplementary Fig. S2).

### Sample binding analyses: Fab NC41–influenza virus neuraminidase N9 and Fab 44.1–hen egg-white lysozyme complexes

The main metrics of interest are the rank of the cognate complex relative to the non-cognate complexes and the magnitude of the predicted binding scores. We used Rosetta’s docking interface score^19^ (based on the Talaris2013 force field^20^) to rank the complexes. Rosetta interface score is defined as ${\rm{Isc}}={E}_{{\rm{bound}}}-{E}_{{\rm{unbound}}}$, where ${E}_{{\rm{bound}}}$ is the score of the bound complex and ${E}_{{\rm{unbound}}}$ is the sum of the scores of the individual protein partners in isolation. The score function is a linear combination of several score terms including a Lennard–Jones potential, an implicit solvation potential^21^, an orientation-dependent hydrogen bonding potential^22^, a Coulomb electrostatic potential with a distance-dependent dielectric, a side-chain torsional potential^23^, and a knowledge-based residue pair term based on the probability of proximity of two amino acids in the PDB^24^.

**Figure 2:**
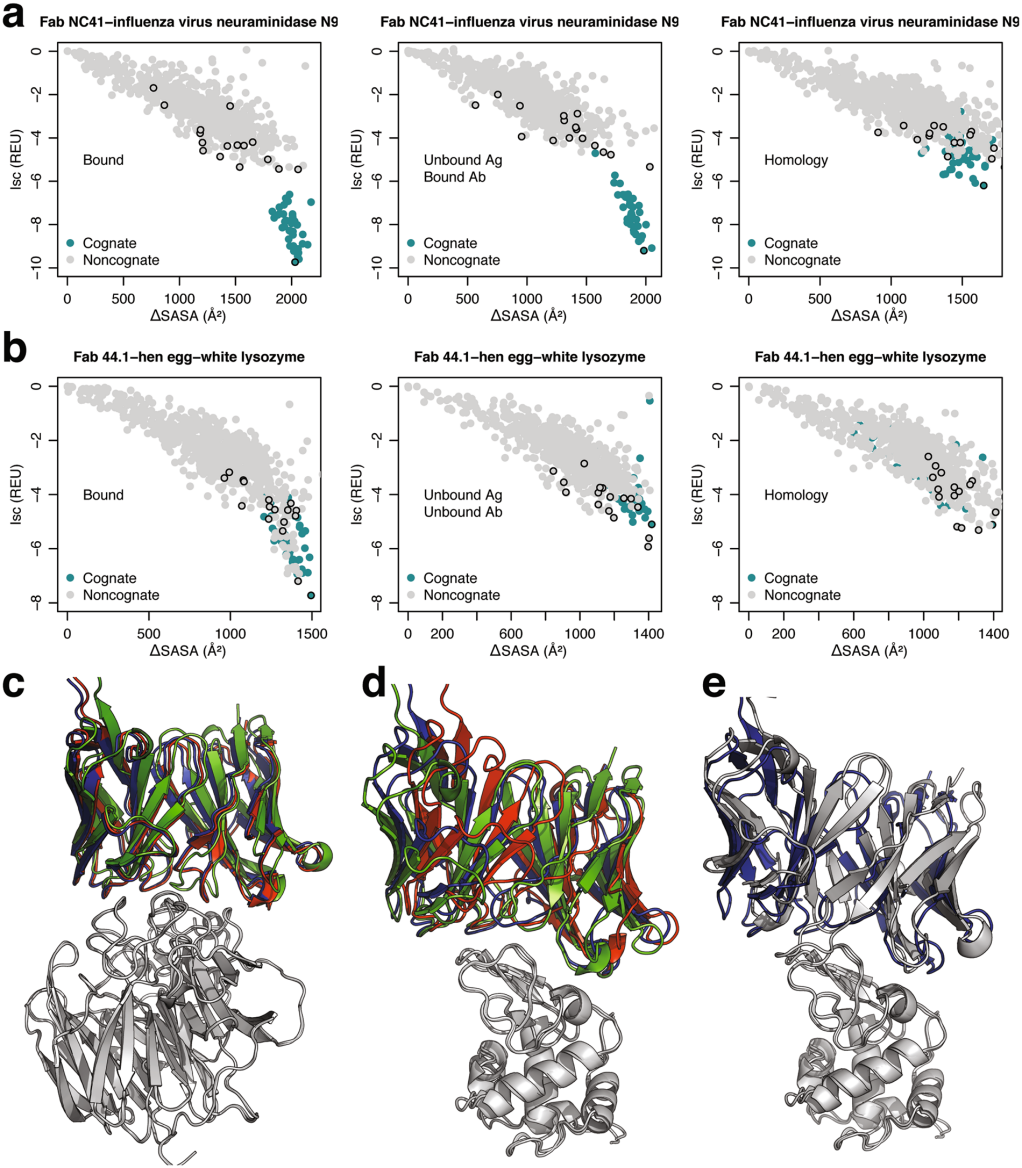
Binding discrimination in Fab NC41–influenza virus neuraminidase N9 and Fab 44.1–hen egg-white lysozyme complexes. Surface area change upon binding (ΔSASA) vs. Interface scores for model structures in antibody cross-docking tests using bound, unbound, and homology-modeled backbones in (**a**) influenza virus neuraminidase N9, and (**b**) hen egg-white lysozyme. Sixteen non-cognate structures (grey) are compared to cognate structures (turquoise); black outline indicates the top-scoring model for each antibody–antigen pair. The top-scoring cognate complexes generated using bound (red), unbound (blue), and homology (green) complexes in (**c**) influenza virus neuraminidase N9, and (**d**) hen egg-white lysozyme complexes. (**e**) Top-scoring cognate (blue) and non-cognate (grey) antibody against hen egg-white lysozyme.

Figure 2 compares the interface scores for antibody complexes involving influenza virus neuraminidase N9 (Cognate Fab NC41; 1NCA) and hen egg-white lysozyme (Cognate Fab 44.1; 1MLC) generated using the local refine routine (Fig. 2a,b). Interface scores for the cognate and non-cognate complexes (turquoise and grey, respectively; top-scoring complexes for each antibody against the target antigen are highlighted with a black outline) generated starting from bound, unbound, and homology backbones are plotted as a function of the complex interface size (ΔSASA in Å^2^). In general, interface scores are lower (better) for larger candidate interfaces, and the cognate antibody–antigen complexes have both larger interfaces and lower scores. In both the antigens, the calculated energy gap between the cognate and non-cognate antibodies is small, even though the non-cognate complexes likely bind weakly or not at all.

In influenza virus neuraminidase N9, interface scores of the top-scoring cognate complexes (Fig. 2a) drop from −9.7 to −9.2, and further to −6.2 REU when using bound antibody/antigen, unbound antigen/bound antibody, and unbound antigen/homology antibody backbones, respectively, even though all the complexes involve the same epitope (Fig. 2c). The cognate scores remain better than the corresponding interface scores of all the top-scoring non-cognate complexes in each category (−5.5, −5.3, and −5.8 REU, respectively). In hen egg-white lysozyme, interface scores of the top-scoring cognate complexes (Fig. 2b) are −7.7, −5.1, and −5.1 REU when using antibody bound, unbound, and homology backbones against the same epitope (Fig. 2d). Interface score can discriminate the cognate antibody using bound models (top non-cognate score: −7.2 REU), but other non-cognate antibodies surpass even the top-scoring cognate antibody when using unbound and homology models. For example, when using unbound backbones, the top-scoring non-cognate complex generated using the antibody from the Fab HC19–influenza hemagglutinin T131I complex (2VIS) scores better (−5.9 REU) than the top-scoring cognate complex (−5.1 REU) even though both antibodies target the same lysozyme epitope (Fig. 2e). Finally, even the top-scoring bound cognate interface scores of −9.7 and −7.7 REU in the two complexes underestimate the true binding affinities (~11 and 9.6 kcal/mol, respectively, mapping^13, 25, 26^ REUs to kcal/mol).

In summary, interface score distinguishes the cognate antibody from the non-cognate antibodies in both the cases when using the bound antibody coordinates but fails in the case of Fab 44.1–hen egg-white lysozyme complex when using unbound and homology-modeled backbones, likely due to the decrease in the accuracy of the antibody backbone conformations used for generating the models.

### Antibody backbone accuracy is critical for accurate binding partner identification

**Figure 3:**
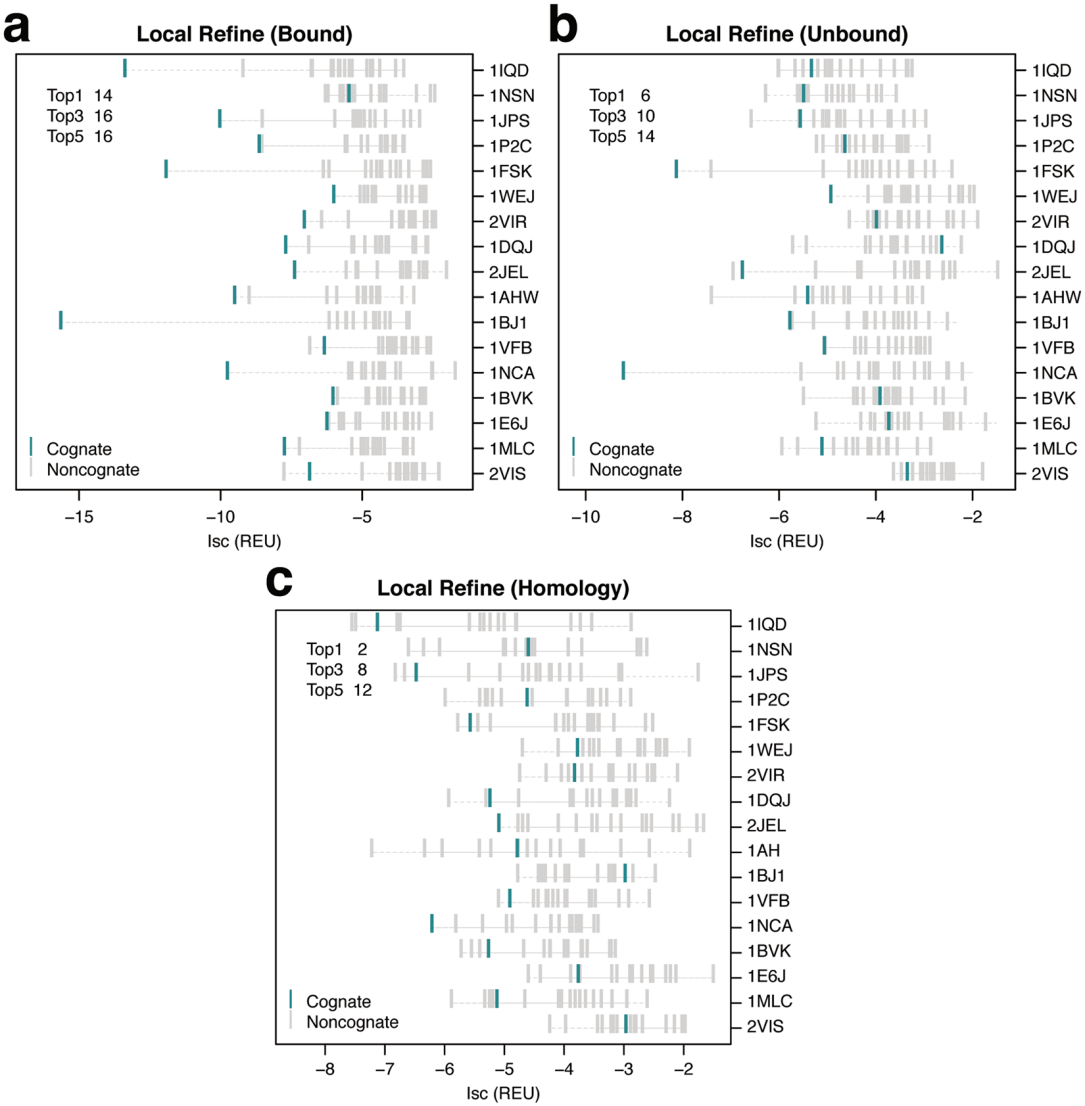
Ranks of cognate complexes among local refine antibody–antigen models. Interface scores (Isc) of the top-scoring cognate (turquoise) and non-cognate (grey) local refine models for (**a**) bound, (**b**) unbound, and (**c**) homology antibody–antigen complexes. Each row indicates a single antigen represented by the PDB ID of the bound complex. Antigens are sorted based on decreasing experimental binding affinities for their native antibodies (top to bottom). The top left corner shows the number of antigens where the cognate antibody complex is ranked in the top 1, 3, and 5 top-scoring cognate and non-cognate models generated for the antigen.

We used interface scores (Isc) to rank the cognate and non-cognate models for each antibody–antigen pair in the dataset. Figure 3 shows the interface scores of the top-scoring cognate and non-cognate models for each antigen. Using bound backbones (Fig. 3a), the cognate antibody–antigen pair is the top-scoring model in 14/17 antigens, and it is one of the top three scoring models in 16/17 antigens. Using unbound backbones, the cognate pairs ranked at the top in six targets, and in the top three in ten targets (Fig. 3b), compared to two and eight cognate pairs at the top and top three when using homology backbones (Fig. 3c). Four of the six top-ranked cognate pairs using unbound backbones are from targets missing unbound antibody structures and were generated using bound antibody coordinates. The drop in the prediction accuracy correlates with the drop in the accuracy of the backbone coordinates moving from bound to unbound, and eventually homology complexes. The decline in the discrimination capability is not surprising as backbone conformation is a critical determinant of protein–protein docking accuracy^27^. Inaccuracies in the backbone often translate to docking errors through sub-optimal arrangement of the interface residues in the docking models.

**Figure 4:**
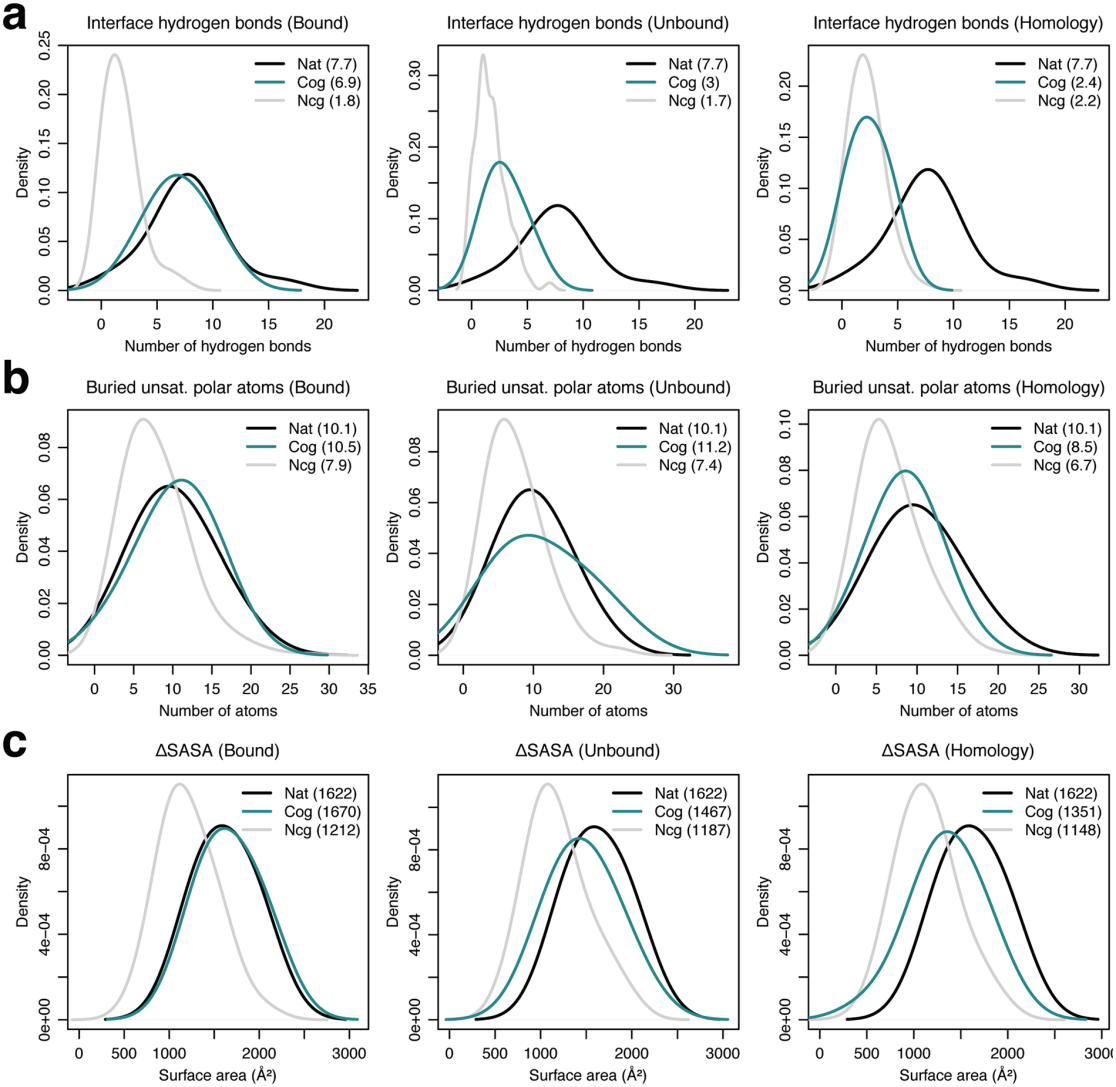
Interface metric distributions for local refine models. Kernel density estimate curves for the number of (**a**) interface hydrogen bonds, (**b**) buried unsatisfied interface polar atoms, and (**c**) surface area change upon binding (ΔSASA) for the native crystal complexes (black), and the top-scoring cognate (turquoise) and non-cognate (grey) models generated using local refine for bound, unbound, and homology antibody–antigen complexes.

To assess the interface quality of the top-scoring cognate and non-cognate models, we first calculated the distribution of the number of antibody–antigen interface hydrogen bonds. As shown in Fig. 4a, the native crystal complexes contain 7.7 ± 3.3 interface hydrogen bonds, compared to 6.9 ± 2.4 and 1.8 ± 1.7 hydrogen bonds in the top-scoring cognate and non-cognate models when using bound backbones. The average number of interface hydrogen bonds drop to 3 ± 1.5 and 1.7 ± 1.4 for the top-scoring cognate and non-cognate models using unbound backbones, respectively, and finally 2.4 ± 1.5 and 2.2 ± 1.5 with homology-modeled antibody backbones. The number of interface hydrogen bonds in the top-scoring cognate models thus decline moving from bound to unbound, and homology complexes, but are similar for the top-scoring non-cognate models across the three categories. As the number of interface hydrogen bonds in cognate complexes drop, there is a greater chance the resulting buried unsatisfied polar atoms contribute to unfavorable interfaces affecting the discrimination power of the interface score.

We computed the number of buried *unsatisfied* polar atoms (ignoring contribution of structural waters) across the interfaces of the top-scoring cognate and non-cognate models. As shown in Fig. 4b, on average, crystal structures of the antibody–antigen complexes have 10.1 ± 4.3 buried unsatisfied polar atoms at the interface, with the top-scoring cognate and non-cognate bound models containing 10.5 ± 4.2 and 7.9 ± 4.2 buried unsatisfied interface polar atoms, respectively. The number of buried unsatisfied interface polar atoms in the top-scoring cognate and non-cognate complexes remain about the same at 11.2 ± 5.7 and 7.4 ± 4.1 when using unbound backbones, but fall to 8.5 ± 3.7, 6.7 ± 3.6, respectively, with homology-modeled antibody backbones. Surprisingly, the number of buried unsatisfied interface polar atoms in the top-scoring cognate complexes are always higher than the corresponding non-cognate complexes in each category. In addition, the reported drop in the number of interface hydrogen bonds between bound and unbound backbones does not increase the number of buried unsatisfied interface polar atoms (which remain about the same). Finally, the number of buried unsatisfied interface polar atoms drop from unbound to homology models correlating with a drop in the number of hydrogen bonds, more so in the top-scoring cognate models. One reason for this unexpected behavior is a simultaneous decline in the absolute interface sizes between i) bound, unbound, and homology, and ii) cognate and non-cognate docking models.

Finally, we investigated the interface sizes of the generated antibody–antigen complexes. Figure 4c shows the surface area change upon binding (ΔSASA) in the top-scoring cognate and non-cognate models. The average ΔSASA in the crystal complexes is 1622 ± 290 Å^2^, and it is 1670 ± 299 Å^2^ and 1212 ± 317 Å^2^ in cognate and non-cognate models, respectively, generated using bound backbone coordinates. ΔSASA in the cognate and non-cognate models is 1467 ± 321 Å^2^ and 1187 ± 332 Å^2^ with unbound backbones, and finally, 1351 ± 337 Å^2^ and 1148 ± 322 Å^2^ using homology backbones. The interface sizes in the top-scoring cognate models decrease using bound, unbound, and homology backbones, but remain roughly the same in the top-scoring non-cognate models. This difference in the interface size trends between cognate and non-cognate models explains the drop in the number of interface hydrogen bonds across cognate but not non-cognate models, and the sharp drop in the number of buried unsatisfied polar atoms in the top-scoring cognate models.

In summary, the interface quality of the top-scoring cognate models declines when using bound, unbound and eventually homology backbones to model the complexes, while the interface quality of the top-scoring non-cognate models expectedly remains about the same (see Table 2 for summary). Since the structures generated by local refine comprehensively sample the antibody–antigen orientation space around the starting epitope, the decline in the prediction accuracy is primarily due to the inaccuracies in the backbone conformations in unbound and homology models.

**Table 2:** Interface metrics for cognate and non-cognate models generated using local refine and local dock.

Method	Metric	Crystal structure	Bound		Unbound		Homology	
			Cognate	Non-cognate	Cognate	Non-cognate	Cognate	Non-cognate
Local Refine	Interface hydrogen bonds	7.7 ± 3.3	6.9 ± 2.4	1.8 ± 1.7	3 ± 1.5	1.7 ± 1.4	2.4 ± 1.5	2.2 ± 1.5
	Buried unsat. interface polar atoms	10.1 ± 4.3	10.5 ± 4.2	7.9 ± 4.2	11.2 ± 5.7	7.4 ± 4.1	8.5 ± 3.7	6.7 ± 3.6
	Surface area change upon binding (Å2)	1622 ± 290	1670 ± 299	1212 ± 317	1467 ± 321	1187 ± 332	1351 ± 337	1148 ± 322
Local Dock	Interface hydrogen bonds	7.7 ± 3.3	6 ± 3	3.2 ± 1.8	3.1 ± 2.1	3.4 ± 1.8	3 ± 1.6	3.2 ± 1.9
	Buried unsat. interface polar atoms	10.1 ± 4.3	11.7 ± 3.7	8.4 ± 3.7	9.2 ± 4	8.3 ± 3.5	8.4 ± 3.5	7.8 ± 3.3
	Surface area change upon binding	1622 ± 290	1636 ± 290	1309 ± 258	1394 ± 316	1328 ± 251	1332 ± 213	1292 ± 265

### Expanding antibody–antigen model diversity reduces binding discrimination

The cognate and non-cognate models generated by the local refine protocol are typically under 5 Å C_α_ Irmsd from the native antibody–antigen complexes. That is, model diversity is limited because most model structures have interfaces around the epitope of the antigen. The limited diversity can hinder generation of native-like complexes with tightly-packed interfaces in non-cognate complexes or in cognate complexes where the homology models are not very accurate. To address this, we increased rigid-body sampling diversity using the standard RosettaDock local dock routine. Using local dock, we generated 1000 models starting from a random structure picked from the local refine ensemble for each antibody–antigen pair. In local dock, the starting structures are perturbed by about 3 Å translation and 8° rotation around the axis joining the centers of the two partners generating models up to 20 Å C_α_ Irmsd from the starting complex.

**Figure 5:**
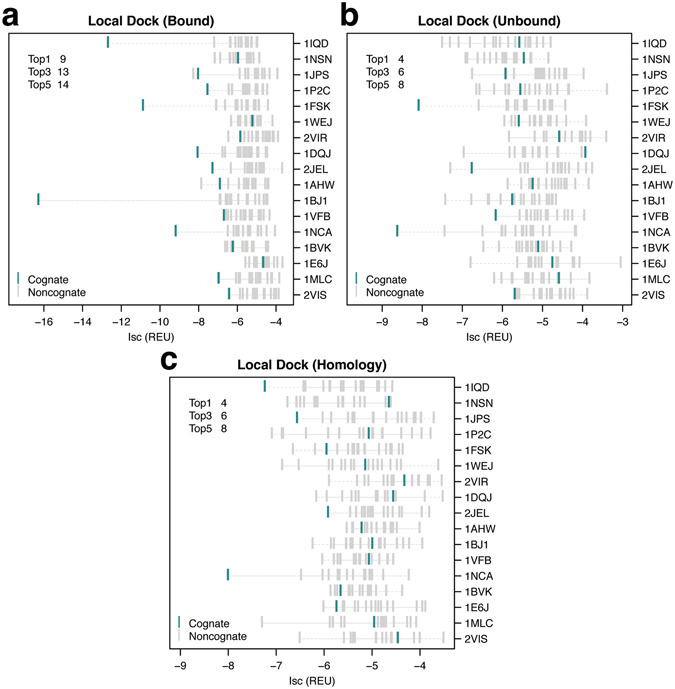
Ranks of cognate complexes among local dock antibody–antigen models. Interface scores (Isc) of the top-scoring cognate (turquoise) and non-cognate (grey) local dock models for (**a**) bound, (**b**) unbound, and (**c**) homology antibody–antigen complexes. Each row indicates a single antigen represented by the PDB ID of the bound complex. Antigens are sorted based on decreasing experimental binding affinities for their native antibodies (top to bottom). The top left corner shows the number of antigens where the cognate antibody complex is ranked in the top 1, 3, and 5 top-scoring cognate and non-cognate models generated for the antigen.

We compared the interface scores of the top-scoring cognate and non-cognate models for each antigen (Fig. 5). Using interface scores, the cognate antibody–antigen pair is the top-scoring model in nine of the 17 target antigens, and is one of the top three scoring models in 13/17 targets using bound backbones to generate the complexes (Fig. 5a). The cognate pairs ranked at the top in four targets, and in the top three in six targets using both unbound and homology models (Fig. 5b,c). Two of the four top-ranked cognate pairs in unbound models are from targets missing unbound antibody structures and thus generated using bound antibody coordinates. As expected, the number of correctly identified cognate antibody–antigen pairs using local dock is lower than local refine when using bound, unbound backbone coordinates. Encouragingly, when using homology complexes, local dock places more cognate pairs at the top (four) compared to local refine (two).

**Figure 6:**
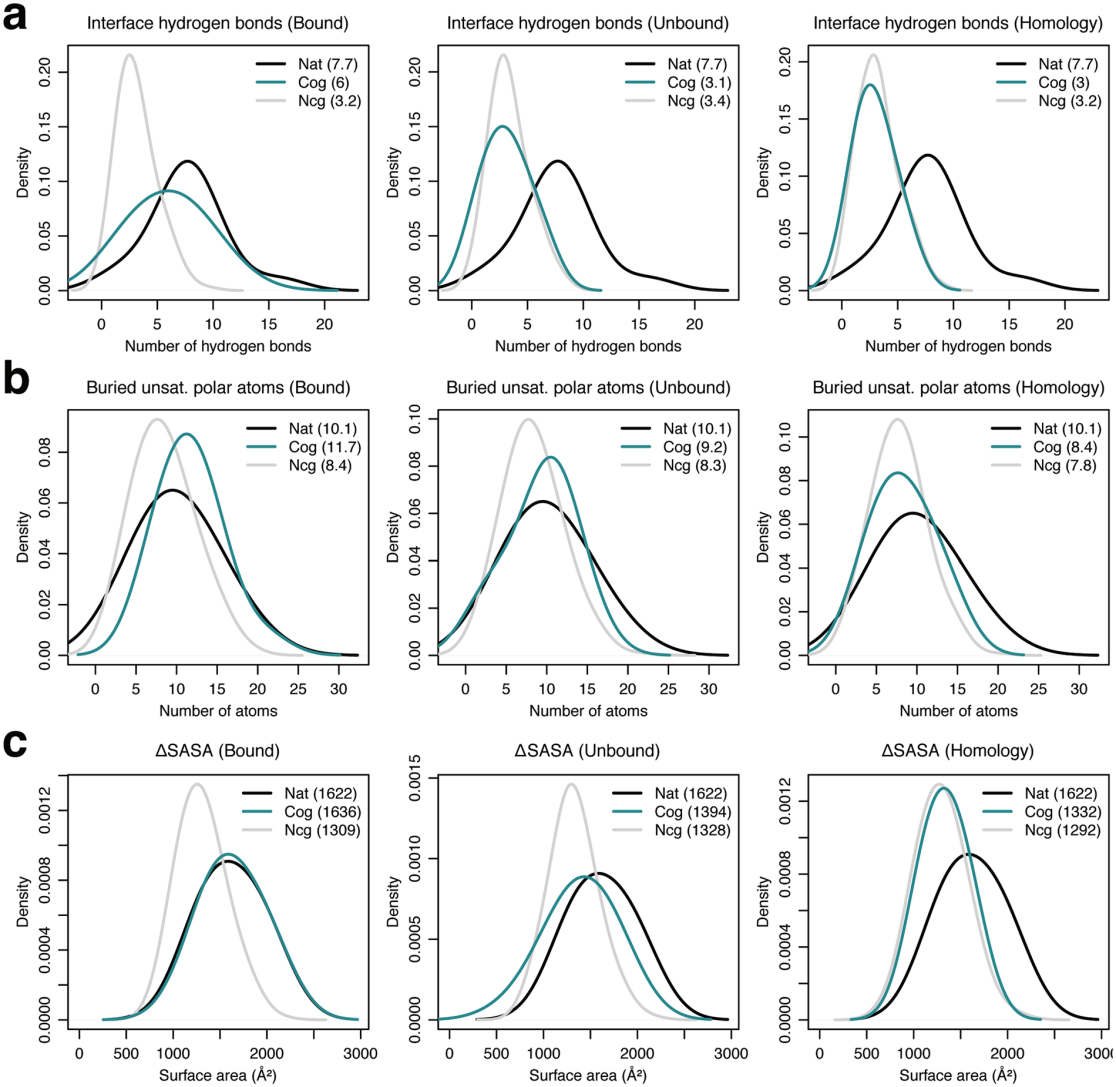
Interface metric distributions for local dock models. Kernel density estimate curves for the number of (**a**) interface hydrogen bonds, (**b**) buried unsatisfied interface polar atoms, and (**c**) surface area change upon binding (ΔSASA) for the native crystal complexes (black), and the top-scoring cognate (turquoise) and non-cognate (grey) models generated using local dock for bound, unbound, and homology antibody–antigen complexes.

We again calculated distributions of the three interface quality metrics: (i) number of interface hydrogen bonds, (ii) number of buried unsatisfied interface polar atoms, and (iii) surface area change upon binding for the top-scoring cognate and non-cognate models generated by local dock (see Table 2 for summary). The top-scoring cognate models generated starting from bound backbones contain 6 ± 3 interface hydrogen bonds, 11.7 ± 3.7 buried unsatisfied interface polar atoms, and 1636 ± 290 Å^2^ ΔSASA, compared to 3.2 ± 1.8 hydrogen bonds, 8.4 ± 3.7 unsatisfied atoms, 1309 ± 258 Å^2^ ΔSASA, respectively, in the top-scoring non-cognate models (Fig. 6). Using unbound backbones, cognate and non-cognate models contain 3.1 ± 2.1 hydrogen bonds, 9.2 ± 4 unsatisfied atoms, 1394 ± 316 Å^2^ ΔSASA, and 3.4 ± 1.8 hydrogen bonds, 8.3 ± 3.5 unsatisfied atoms, 1328 ± 251 Å^2^ ΔSASA, respectively. Finally, the top-scoring models cognate and non-cognate models generated using homology backbones contain 3 ± 1.6 hydrogen bonds, 8.4 ± 3.5 unsatisfied atoms, 1332 ± 213 Å^2^ ΔSASA, and 3.2 ± 1.9 hydrogen bonds, 7.8 ± 3.3 unsatisfied atoms, 1292 ± 265 Å^2^ ΔSASA, respectively. The interface quality of the cognate models generated by local dock drops drastically moving from bound backbones, but is similar across models generated using unbound and homology backbones correlating with the same trend observed in the binding discrimination performance. For the non-cognate models, the average interface quality is similar across all the three categories.

In general, the binding discrimination performance in local dock generated models is lower compared to the models generated using local refine because the greater model diversity increases the chance of false positive interfaces picked up by the score function. One exception is models generated from homology-modeled antibody backbones where the greater antibody–antigen orientation diversity in local dock compensates for the backbone inaccuracies resulting in higher quality interfaces in both cognate and non-cognate models. Local dock achieves more top-ranked cognate complexes compared to local refine using homology-modeled antibodies.

### Effect of experimental binding affinities and antibody backbone modeling errors

**Figure 7:**
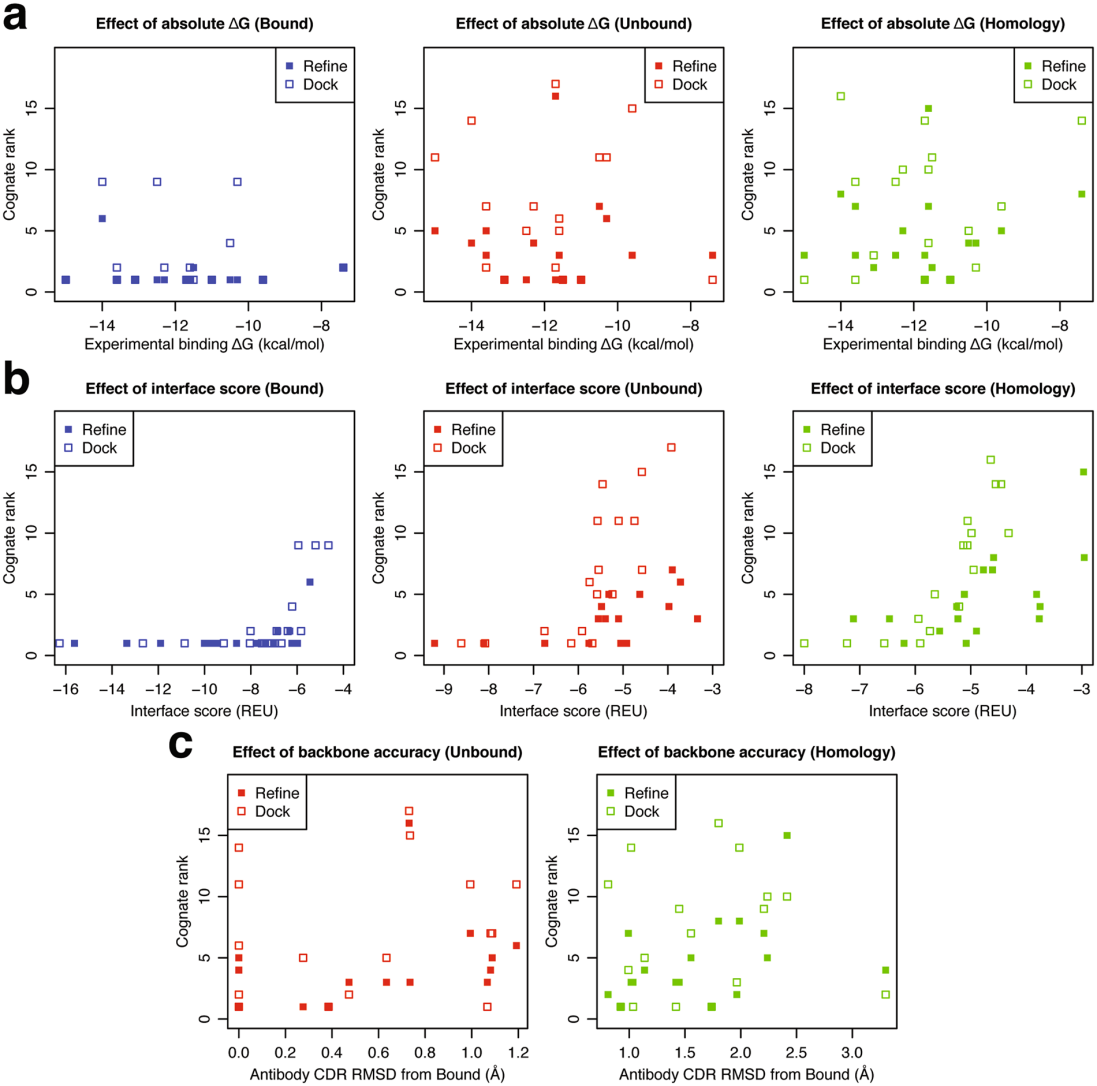
Effects of experimental binding affinities, interface scores, and antibody backbone accuracy. Correlation plots comparing ranks of the top-scoring cognate models to (**a**) experimental binding affinities (ΔG), (**b**) interface scores, and (**c**) antibody CDR backbone accuracy for bound (blue), unbound (red), and homology (green) complexes. Models generated using local refine and local dock are shown as filled and empty squares, respectively.

To determine the effect of absolute binding affinities of the antibody–antigen complexes on the binder predictions, we plotted correlations of the ranks of the top-scoring cognate models vs. the absolute experimental ΔG values of the native antibody–antigen complexes. As shown in Fig. 7a, binder discrimination of the models generated using local refine and local dock is independent of the absolute binding affinities for bound, unbound, and homology complexes. For example, the cognate antibody against Factor VIII domain C2, Fab B02C11 (PDB: 1IQD) which is the stronger binder in the dataset (experimental *K*_d_ < 0.014 nM) ranks first both in local refine and local dock against its antigen when using bound backbone, but ranks fifth and eleventh, respectively, when using unbound backbone, and third and first when using homology-modeled backbone. The weakest binder in the dataset, Fab HC19 against influenza virus hemagglutinin T131I mutant (2VIS), ranks second both in local refine and local dock when using bound, but ranks third and first when using unbound, and eighth and fourteenth when using homology-modeled backbones. Therefore, when using unbound backbones, prediction accuracy is higher in the case of the weakest binder compared to the strongest binder in the dataset. It is not surprising binding discrimination is not strongly dependent on absolute binding affinities of the native antibody–antigen complexes, as the score function is not calibrated for absolute binding affinities but to distinguish native vs. non-native interfaces.

We next tested the dependence of the ranks of the cognate complexes on their interface scores (Fig. 7b). The cognate ranks are good (rank 1 or 1–3) when interface scores Isc < −6 REU (*i.e*. tight binders), and cognate rank quality decreases (higher ranks) for Isc above −6 REU. The increase in the cognate rank is especially evident in unbound and homology complexes where only three complexes from the dataset have Isc < −6 REU, compared to bound complexes where 76%, 88% of the complexes have Isc < −6 REU when using local refine, lock dock, respectively. Since interface score is a measure of the complex interface quality, the increase in the cognate rank highlights the drop in the interface quality when using unbound and homology backbones.

We also calculated the effect of antibody backbone accuracy on binding discrimination by plotting correlations of the ranks of the top-scoring cognate complexes vs. RMSD of the antibody CDRs from the bound backbone coordinates (Fig. 7c). As expected, binder discrimination accuracy in general drops at higher CDR RMSDs. Specifically, when using local refine with unbound and homology-modeled backbones, the cognate rank progressively worsens with increasing antibody CDR RMSD. The weak correlation with the antibody backbone accuracy in local refine but not local dock hints that the limited antibody–antigen orientation diversity in local refine is inadequate to compensate for the backbone errors. This observation is consistent with the small increase in the number of top-scoring cognate models when using local dock (vs. local refine) in models generated using homology-modeled backbones.

Finally, we evaluated the impact of the deviation of the final top-scoring cognate and non-cognate complexes from the original cognate crystal complexes on binding discrimination for the local refine and local dock cases. Supplementary Fig. S3 shows the antibody ligand-RMSD (Ab_L_RMSD, see Methods) versus the rank of all cognate and non-cognate top-scoring models for all 17 targets and various docking cases. Like the CDR RMSDs, it is necessary for the antibody ligand-RMSD to be small for the cognate antibody to have a top rank relative to the non-cognate antibodies. Conversely, top-ranked non-cognate antibody complexes are sometimes close to the cognate complex structure (Ab_L_RMSD < 5 Å), and sometimes quite different (up to ~30 Å).

### Binder discrimination summary: Antibody–antigen complexes are challenging targets

**Figure 8:**
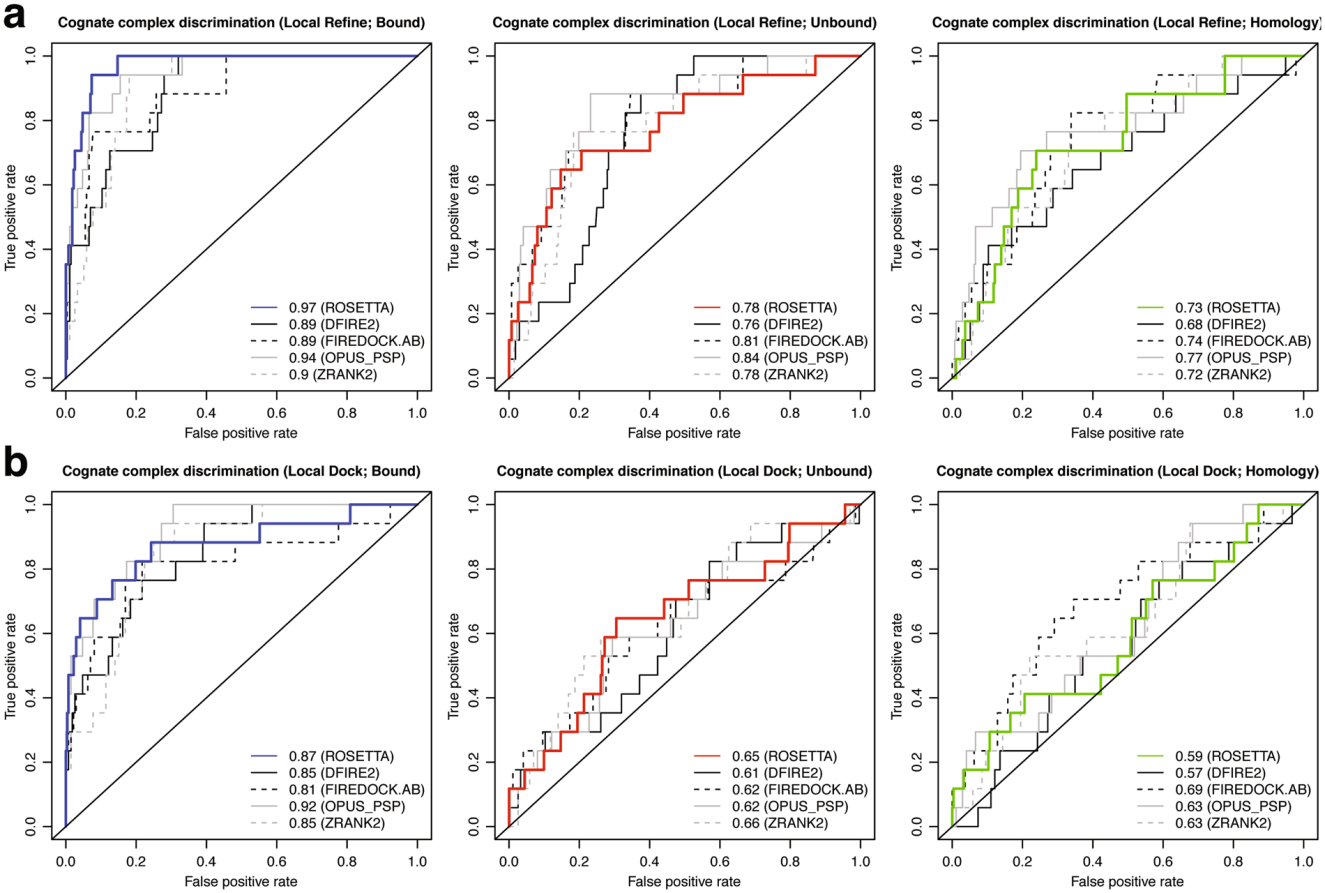
Receiver Operating Characteristic (ROC) curves for binder discrimination. ROC curves using Rosetta interface score of the top-scoring model as the classifier for (**a**) local refine, and (**b**) local dock. The native cognate antibody***–***antigen interaction pairs are true positives. ROC curves computed using four additional potentials: DFIRE2, FIREDOCK.AB, OPUS_PSP, and ZRANK2 are shown for comparison. The bottom right corner of the plot shows the area under the curve (AUC) for Rosetta bound (blue), unbound (red), and homology (green) complexes compared to the other potentials.

To compare the binding discrimination power of the interface score across various categories, we calculated the Receiver Operating Characteristic (ROC) curves using the interface score of the top-scoring model as the classifier in bound, unbound, and homology complexes for both local refine and local dock routines (Fig. 8). We also calculated the ROC curves for the complexes using four other widely-used scoring potentials computed using the CCharPPI^28^ web server: DFIRE2^29^ interaction energy, total FireDock^30^ energy (antibody–antigen energy function), OPUS-PSP^31^ all-atom potential, and ZRANK2^32^ scoring function. We considered the native cognate antibody***–***antigen interaction pairs as the only true positives.

In ROC curves, the area under the curve (AUC) values represent the performance of the scoring potential as a binary binder vs. non-binder classifier. Using Rosetta interface score as the classifier for models generated using the local refine routine, the AUC for the bound complexes is 0.97, followed by 0.78 and 0.73 for unbound and homology complexes, respectively (AUC 0.5 = random; 1 = perfect discrimination, Fig. 8a). For local refine models, Rosetta interface score is the best discriminator in bound complexes, but FireDock and OPUS-PSP potential perform better when using unbound and homology complexes. Using interface score as the classifier in local dock routine, the AUC values for bound, unbound, and homology complexes are 0.87, 0.65, and 0.59, respectively (Fig. 8b). For local dock models, Rosetta interface score is one of the top two discriminators in bound and unbound complexes, behind OPUS-PSP and ZRANK2 potentials, respectively. In homology complexes, FireDock outperforms all the other potentials including the Rosetta interface score. Overall, discrimination by the interface score is better than random in all cases, and as expected, drops with an increase in antibody backbone inaccuracy and/or model diversity.

## Discussion

We have presented the first structure-based cross-docking study focused on discrimination of protein binders from non-binders by identifying native antibody–antigen interaction pairs among cognate and non-cognate complexes. After generating an unbiased dataset of cognate and non-cognate complexes, we used Rosetta interface scores of the top-scoring models to rank the complexes. The binding discrimination results are encouraging when using bound backbones with the cognate pair ranked at the top in more than 80% of the antigen targets, compared to 35% and 12% when using unbound and homology-modeled antibody backbones, respectively. Using RosettaDock’s local dock routine to increase antibody–antigen model diversity helps achieve more top-ranked cognate complexes when using homology-modeled antibody backbones, but decreases binding prediction accuracy in bound and unbound backbone-generated models because of the higher false positive rate.

Interface quality of the cognate models declines between using bound and unbound, homology-modeled antibody backbones to model the complexes, but remains about the same in non-cognate models. The drop in the cognate interface quality and hence binding discrimination highlight the sampling limitations of the current docking algorithms when using unbound and homology modeled backbones. Therefore, accurate modeling of the bound structures starting from the unbound structures or homology models is critical for improving accuracy. Flexible backbone algorithms such as EnsembleDock^19^ and SnugDock^33^ based on the conformer selection (CS) binding models previously shown to help improve interface quality^34^ of the models generated using unbound and homology complexes can be of immense value. However, existing flexible docking algorithms may increase false positive rates as the additional backbone flexibility will also help accommodate the energetically unfavorable interface residues at the epitopes in non-cognate models. Moreover, recent results^27^ show that none of the current backbone flexibility generation algorithms successfully sample the bound conformation in a sizable fraction of proteins. Therefore, improving prediction accuracy will require development of novel flexible backbone sampling approaches to model the bound conformation with score functions specially catered to antibody binder discrimination.

Antibody–antigen cross-docking is the only in silico approach that offers a structural perspective in epitope-targeted antibody screening studies. It is useful to screen antibody binders raised from animal immunization campaigns to pick an epitope-diverse selection of antibodies increasing the chances of *in vivo* efficacy that is often a complex function of various interactions involving the antigen. With the rapid rise in the scale of the computing resources, computational structure-based methods are already playing a role in understanding the biological process of generation of antibody repertoires and how antibodies are selected for recognizing pathogens^6^. Further improvements in binding predictions with unbound and homology-modeled backbones will pave the way for studies parsing antibody sequence repertoires for antigen-specific binders and rational engineering of antibodies to minimize off-target activity. This study is the first step towards development of an efficient structure-based cross-docking framework to support high-throughput experimental antibody pipelines.

## Methods

### Homology modeling

Rosetta’s antibody homology modeling protocol, RosettaAntibody 3.0^18^ is used for constructing homology models for the antibodies in the dataset. V_L_,V_H_ homologs with more than 80% sequence identity, and CDR homologs with more than 98% sequence identity are excluded from the database during the template selection stage. Since the antibodies used in the study are part of the RosettaAntibody database, filtering is necessary to avoid picking structural components from the native structures during modeling. Additionally, the sequence identity cutoffs help simulate homology modeling of newly-determined antibody sequences that do not have existing highly homologous structural templates. The coordinate files of the 17 homology-modeled antibodies are provided as Supplementary Dataset 1.

### Starting complex structure generation

For bound complexes, the crystal structure of the antibody–antigen complex is used as the starting structure. For unbound complexes, the starting structure is generated by superimposing the antibody and antigen unbound structures on the bound crystal complex, and for homology complexes, the antibody homology model and antigen unbound structure on the bound crystal complex.

### Local refine

The local refine routine is derived from RosettaDock’s high-resolution refinement stage. Since superposing the V_L_–V_H_ framework of the non-cognate antibody on the cognate antibody to generate the starting non-cognate structure often results in steric clashes with the antigen, the standard refinement routine moves the antibody away from the epitope to lower the total score of the complex. To generate models focused around the epitope, we created a customized local refine antibody–antigen refinement routine. The routine starts minimization of the antibody–antigen orientation with a score function with a low Van der Waals repulsive weight (*w*_rep_ = 0.02) to ignore starting steric clashes. The weight is gradually ramped up to its full value (*w*_rep_ = 0.186) in increments of 33% each over three minimization cycles allowing sampling of conformations that are otherwise inaccessible due to the high-energy barriers.

### Local dock

We used the standard RosettaDock local docking routine^35^ involving a sequence of low-resolution and high-resolution steps to generate local dock models.

### Metrics

Antibody ligand-RMSD (*Ab_L_RMSD*) of a model antibody–antigen complex is defined as the RMSD between the antibody framework C_α_ atoms (receptor) in the model and reference complexes calculated after aligning the antigen backbones (ligand) in the both the complexes.

$$Ab\_L\_RMSD=\sqrt{\frac{{\sum }^{}{({C}_{\alpha }^{Ab\_Frwk}-{C}_{\alpha }^{Ref\_Ab\_Frwk})}^{2}}{{N}_{Ab\_Frwk\_Res}}}$$

where ${C}_{\alpha }^{Ab\_Frwk},\,{C}_{\alpha }^{Ref\_Ab\_Frwk}$ are the *C*_α_-atom coordinates of the antibody framework residues in the model and reference antibodies, respectively. The RMSDs are calculated over all the antibody framework residues (${N}_{Ab\_Frwk\_Res}$).

Antibody ligand-RMSDs are analogous to the commonly used ligand RMSDs in docking calculations.

### Rosetta command line

The algorithms in the manuscript are implemented using the Rosetta molecular modeling suite. The Rosetta command-line arguments used for the calculations are as follows:

### *Cognate and non-cognate model generation*

- Local refine
  ab_binding.<exe>
  –s 1BVK.1AHW.pdb
  –docking:partners LH_C
  –docking:docking_local_refine
  –pH:pre_process
  –pH:cognate_pdb 1AHW.b.pdb
  –pH:cognate_partners LH_C
  –ex1 –ex2aro –use_input_sc
  –nstruct 50
  where 1BVK.1AHW.pdb is the structure generated after superposing the non-cognate antibody from 1BVK.pdb on the cognate antibody in 1AHW.pdb. The –docking:partners and –pH:cognate_partners arguments identify the receptor and ligand chains in non-cognate and cognate complexes, respectively. –pH:cognate_pdb provides the cognate bound complex.
- Local dock

docking_protocol.<exe>

–s 1BVK.1AHW.pdb –native 1BVK.1AHW.pdb

–dock_pert 3 8 –spin –partners LH_C –ex1 –ex2aro

–nstruct 1000

### Homology modeling

- RosettaAntibody homology modeling

antibody_H3.<exe>

–s 1AHW_h.pdb

–antibody:remodel perturb_kic

–antibody:snugfit true

–antibody:refine refine_kic

–antibody:cter_insert false

–antibody:flank_residue_min true

–antibody:bad_nter false

–antibody:h3_filter false

–antibody:h3_filter_tolerance 5

–antibody:constrain_cter

–antibody:constrain_vlvh_qq

–constraints:cst_file 1AHW_cter_constraint

–loops:legacy_kic false

–loops:kic_min_after_repack true

–loops:kic_omega_sampling

–loops:allow_omega_move true

–kic_bump_overlap_factor 0.36

–loops:ramp_fa_rep –loops:ramp_rama

–loops:outer_cycles 5

–corrections:score:use_bicubic_interpolation false

–ex1 –ex2aro –extrachi_cutoff 0

–nstruct 2000

## Electronic supplementary material

Supplementary Information

Supplementary Dataset 1
