# Supplementary material for: Structure-based cross-docking analysis of antibody–antigen interactions
Source: Sci Rep. 2017 Aug 15;7:8145. doi: 10.1038/s41598-017-08414-y (PMC5557897; doi:10.1038/s41598-017-08414-y)
Supplement: Supplementary file 1 — Supplementary Information [file 41598_2017_8414_MOESM1_ESM.pdf]

Supplementary information

Structure-based cross-docking analysis of antibody–antigen interactions

Krishna Praneeth Kilambi[1,†], Jeffrey J. Gray[1,*]

1.Department of Chemical and Biomolecular Engineering, The Johns Hopkins University, Baltimore, MD 21218, USA

† Current address: Biogen, Cambridge, MA 02142, USA

* Correspondence: jgray@jhu.edu

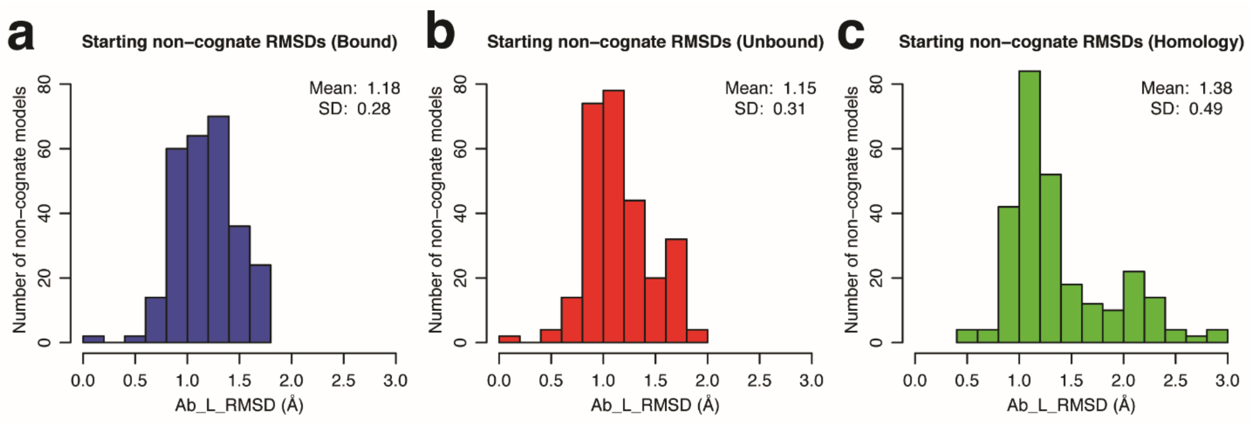

Figure S1: Starting non-cognate antibody deviations. Starting antibody ligand-RMSD distributions of the non-cognate antibodies superimposed on the corresponding cognate antibody in (**a**) bound, (**b**) unbound, and (**c**) homology complexes. Antibody ligand-RMSDs for the antibodies are calculated over the C_α atoms of their framework residues after aligning the antigen backbones and are analogous to the ligand RMSDs using in docking calculations. The average RMSDs range from 1.2–1.4 Å from the starting cognate orientation.

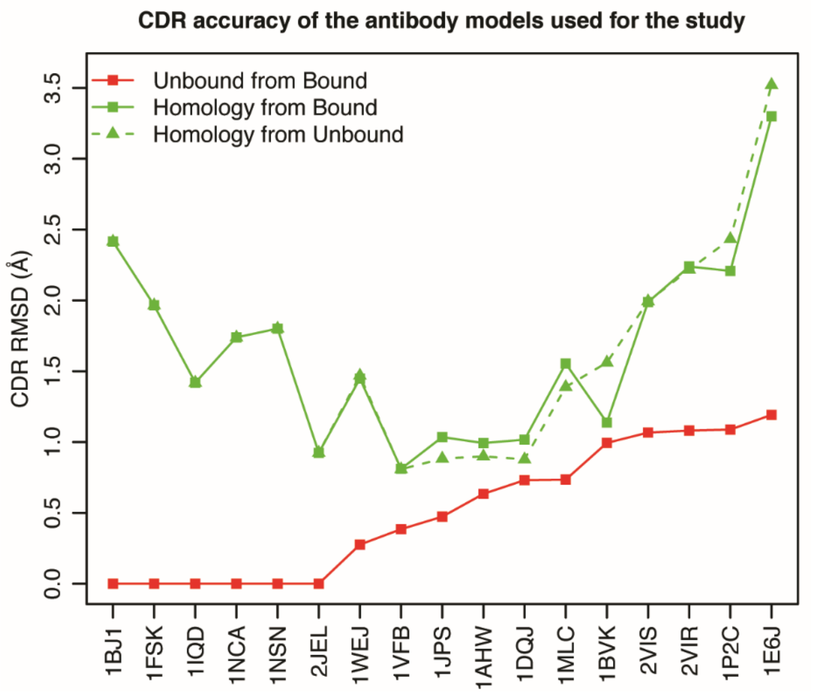

Figure S2: Accuracy of the antibody homology models used for the study. CDR backbone RMSDs of the antibody homology models from their bound (green squares; solid line) and unbound (green triangles; dotted line) backbones. Most of the antibody homology models are closer to their unbound backbones (6/17) than bound backbones (5/17), with the remaining lacking unbound coordinates for comparison. CDR RMSDs of the unbound antibodies compared to their bound backbones (red squares; normal line) are also shown for comparison.

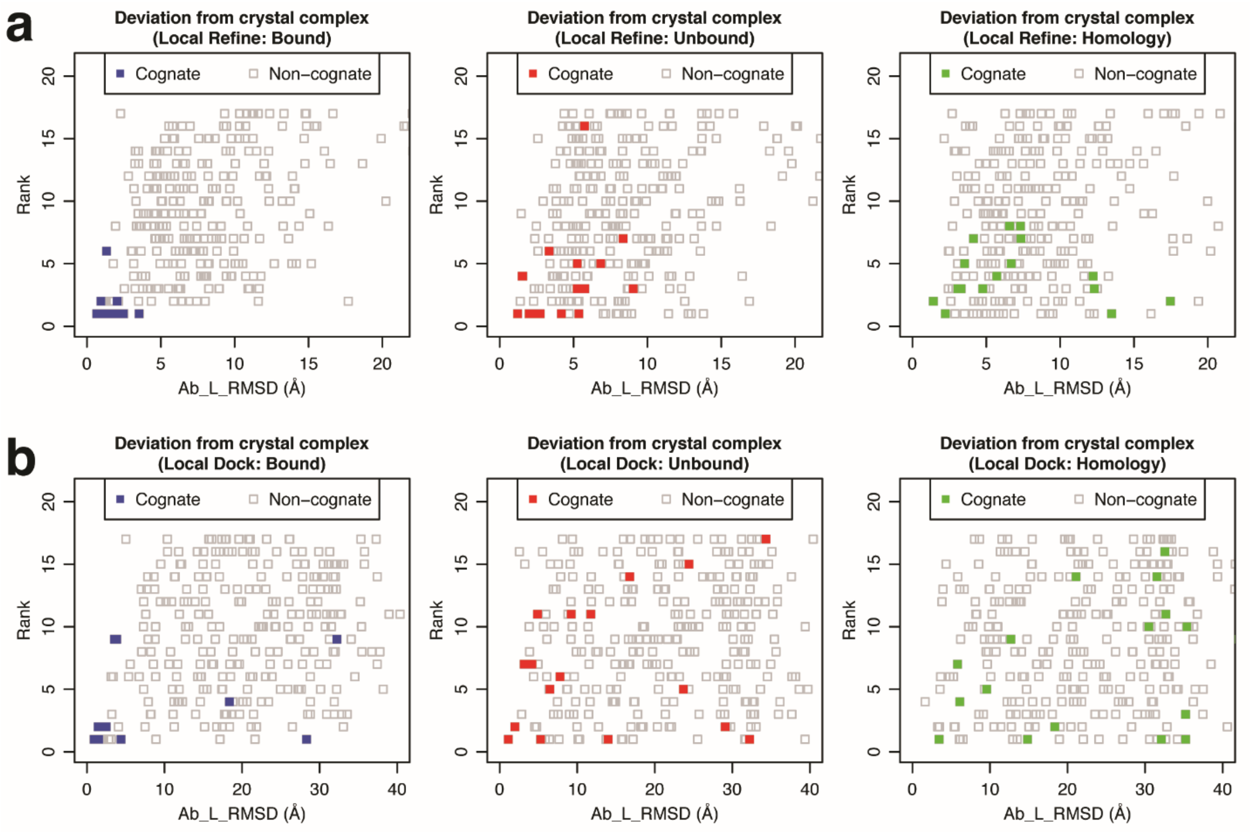

Figure S3: Effects of deviation from the crystal complex orientation on antibody rank. Correlation plots comparing ranks of the top-scoring cognate and non-cognate models and their antibody ligand-RMSDs from the original crystal complex for models generated using (**a**) local refine, and (**b**) local dock for bound (blue), unbound (red), and homology (green) complexes. Antibody ligand-RMSDs for the antibodies are calculated over the C_α atoms of their framework residues after aligning the antigen backbones and are analogous to the ligand RMSDs using in docking calculations. Cognate and non-cognate models (grey) are shown as filled and empty squares, respectively.

Supplemental Dataset 1: Homology models of the 17 antibodies in the test set.
